# Actin Isoform Composition and Binding Factors Fine-Tune Regulatory Impact of Mical Enzymes

**DOI:** 10.3390/ijms242316651

**Published:** 2023-11-23

**Authors:** Jose L. Martin, Aaqil Khan, Elena E. Grintsevich

**Affiliations:** Department of Chemistry and Biochemistry, California State University, Long Beach (CSULB), Long Beach, CA 90840, USA

**Keywords:** actin, isoform, MICALs, NADPH oxidase

## Abstract

Mical family enzymes are unusual actin regulators that prime filaments (F-actin) for disassembly via the site-specific oxidation of M44/M47. Filamentous actin acts as a substrate of Mical enzymes, as well as an activator of their NADPH oxidase activity, which leads to hydrogen peroxide generation. Mical enzymes are required for cytokinesis, muscle and heart development, dendritic pruning, and axonal guidance, among other processes. Thus, it is critical to understand how this family of actin regulators functions in different cell types. Vertebrates express six actin isoforms in a cell-specific manner, but MICALs’ impact on their intrinsic properties has never been systematically investigated. Our data reveal the differences in the intrinsic dynamics of Mical-oxidized actin isoforms. Furthermore, our results connect the intrinsic dynamics of actin isoforms and their redox state with the patterns of hydrogen peroxide (H_2_O_2_) generation by MICALs. We documented that the differential properties of actin isoforms translate into the distinct patterns of hydrogen peroxide generation in Mical/NADPH-containing systems. Moreover, our results establish a conceptual link between actin stabilization by interacting factors and its ability to activate MICALs’ NADPH oxidase activity. Altogether, our results suggest that the regulatory impact of MICALs may differ depending on the isoform-related identities of local actin networks.

## 1. Introduction

Actin is an essential cytoskeletal protein in eukaryotic cells that undergoes reversible monomer-to-polymer (G- to F-actin) transition to exert a diverse array of functions. Mammals express six actin isoforms sharing over 90% sequence similarity: skeletal muscle α-actin, α-smooth muscle actin, α-cardiac actin, γ-smooth muscle actin, cytoplasmic γ-actin, and cytoplasmic β-actin [1]. The biological need for such a multitude of isoforms is unclear since actin’s sequence is highly conserved. Emerging evidence suggests that subtle differences in the sequences and/or dynamics of actin isoforms can be exaggerated upon their interactions with actin-binding proteins and/or posttranslational modifications, potentially leading to a wide range of dynamic behaviors [2,3,4,5].

More than 200 actin regulators are known, but only a few of them have been examined in respect of their actin isoform preference. Mical family enzymes (Molecule Interacting with CasL; MICALs) have the ability to dramatically alter the intrinsic properties of actin filaments and their interactions with binding factors, priming cytoskeletal networks for disassembly in a redox-dependent manner. MICALs are widely expressed and are required for cytokinesis, muscle and heart development, dendritic pruning, and axonal guidance, as well as other key processes in different cells and tissues [6,7]. However, a potential connection between the isoform identity of actin networks in different cell types and the regulatory effects of Mical enzymes is unknown.

**Figure 1:**
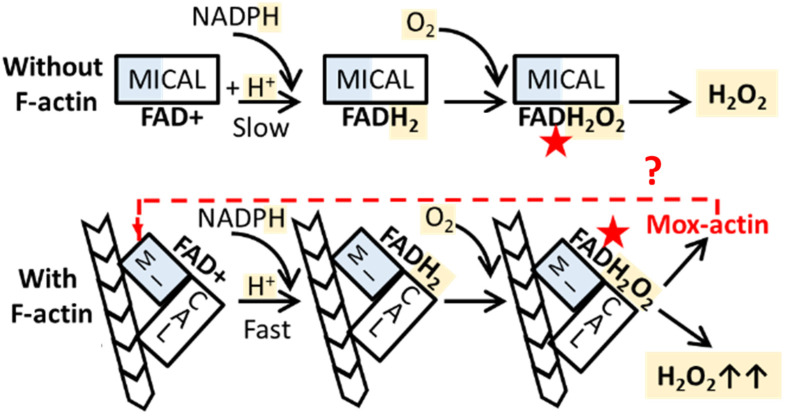
The effects of F-actin on catalytic activity of MICAL enzymes. F-actin acts as a substrate of MICALs and as an activator of their NAPDH oxidase activity. In reductive half-reactions, NADPH serves as an electron donor, reducing FAD+. F-actin induces conformational changes in MICAL structures, facilitating NADPH binding to the enzyme (assuming that the lower Km(NADPH) observed in the presence of F-actin can serve as a measure of pseudo-affinity) and accelerating the FAD+ reduction rate (thick black arrow, lower panel) [8,10,11]. Reduced FADH_2_, in turn, reacts with molecular oxygen (O_2_) to form an unstable C4a-hydroperoxylflavin intermediate (red stars in the schematic), which either is utilized for the oxidation of primary substrates such as F-actin (lower panel, Mox-actin is produced) or decays with the generation of hydrogen peroxide (H_2_O_2_, upper and lower panels). As a result, an increased Mical turnover rate (kcat(NADPH)) was observed in the presence of F-actin. The potential of Mox-actin as an activator of the NADPH oxidase activity of MICALs was investigated in this work (lower panel, dashed red arrow and a question mark).

The enzymatic activity of MICALs requires the presence of the prosthetic group (FAD+), NADPH (coenzyme), and molecular oxygen (Figure 1). These enzymes have postulated monooxygenase and NADPH oxidase activities [8,9,10,11], which are connected through the formation of a common reactive intermediate—C(4a)-hydroperoxyflavin. C(4a)-hydroperoxyflavin can be utilized for the oxidation of primary substrates (molecules transformed in the catalytic sites of these enzymes) or decay with the formation of H_2_O_2_, which (upon its release from the catalytic site) can oxidize secondary targets (Figure 1).

The monooxygenase activity of MICALs results in the site- and stereospecific oxidation of primary substrates, as seen with F-actin [12], calmodulin-kinase II [13], and Arp3b [14]. In F-actin, these enzymes introduce the site- and stereospecific oxidation of M44 and M47 in the self-interacting interface. Such posttranslationally modified actin is referred to as Mox-actin herein. F-actin oxidation by MICALs promotes its nucleotide-bound state-dependent disassembly [15]. In the absence of actin monomers, ADP- (but not ADP-Pi) bound Mox-actin is intrinsically unstable and undergoes catastrophic collapse (depolymerization rates > 80 subunits/s). Structural work revealed that such behavior of ADP-Mox-actin can be explained by the alteration of the protomer–protomer contacts upon Mical-mediated oxidation. Specifically, the oxidation of M44 results in the reduced hydrophobicity of actin’s DNAse I-binding loop (D-loop), which impairs its complementarity to the hydrophobic cleft of an adjacent protomer. Furthermore, the oxidation of M47 results in its bonding to T351 of the adjacent protomer stabilizing the D-loop conformer, which is not observed in the unoxidized F-actin structure. Together, these changes in ADP-Mox-F-actin promote its intrinsic instability [15].

In the presence of ATP-bound monomers, the barbed ends of aged (predominantly ADP-bound) Mox-actin are protected by ATP-/ADP-Pi-bound terminal subunits, and such filaments are less likely to collapse catastrophically, which allows the study of Mox-actin forms [15]. This redox modification of actin also synergizes with other key actin regulators. For instance, the oxidation of M44/M47 improves the binding of an essential actin-severing protein—cofilin—to F-actin, inducing the rapid disassembly of ADP- as well as ADP-Pi-rich (young) actin filaments, which are normally cofilin-resistant [16]. It was also documented that phosphorylated (inactive) cofilin is also capable of severing Mox-actin filaments [17]. Such dismantling of Mox-F-actin is further amplified by the actin-sequestering protein profilin, which promotes Mox-actin disassembly and inhibits its re-incorporation into actin networks by formins and Eva/VASP [18].

The available data are indicative of the evolutionary conservation of actin regulation by MICALs: Micals from *Drosophila melanogaster* (dMical) and three human isoforms (hMical-1/2/3) introduce the same site- and stereospecific (M44/M47) modifications in actin [12,19,20]. Likewise, Mical enzymes can oxidize M44/M47 in different actins (skeletal α-actin, human cytoplasmic, Drosophila melanogaster 5C, and yeast) [12,15,16,20]. However, the functional impact of this oxidation on different actin isoforms remains poorly characterized.

The cellular role of the NADPH oxidase activity of MICALs is not well understood, and it is an emerging area of interest. Some of the FAD-containing enzymes adopt the evolutionary design of a catalytic site, which prevents the elimination of reactive oxygen species for protection from oxidative stress [21]. The fact that MICALs can generate basal levels of H_2_O_2_ in the absence of primary substrates suggests that such activity is biologically relevant. In line with this, the NADPH oxidase activity of MICALs was proposed to regulate the protein function of CRMP [22] and Tau [23] via the H_2_O_2_-mediated oxidation of cysteines. Additionally, Mical activation was linked to the increased generation of reactive oxygen species in some cancers [24,25].

Strikingly, F-actin serves not only as a substrate of Mical enzymes (see above) but also as an activator of their NADPH oxidase activity, resulting in enhanced H_2_O_2_ generation (Figure 1). It is important to note that the connection between actin oxidation by MICALs and actin’s ability to serve as an activator of H_2_O_2_ generation is not well understood. To date, it is not known whether this activation depends on the actin isoform composition and its redox state (unoxidized vs. Mical-oxidized). Importantly, Mical enzymes are expressed in a variety of human tissues, which prompted our investigation of the effects of Mical-induced oxidation on different actin isoforms, as well as a description of their role as activators of the NADPH oxidase activity of these enzymes.

## 2. Results

### 2.1. Mical-Induced Oxidation Differentially Impacts the Intrinsic Stability of Muscle and Cytoplasmic Actins

**Figure 2:**
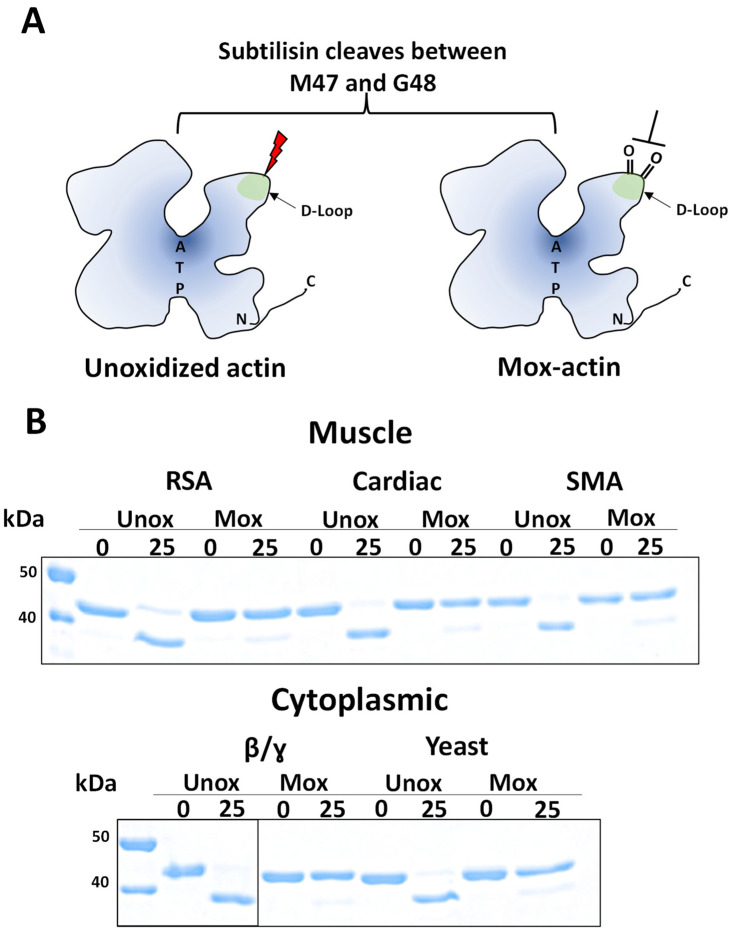
Cytoplasmic and muscle actin forms are substrates of Mical enzymes. (**A**) Limited proteolysis with subtilisin [16] was used to confirm the efficiency of actin oxidation by Mical. Schematic representation of actin cleavage by subtilisin. Subtilisin cleaves (red lightning bolt) between M47 and G48 located in the DNase I-binding loop (D-loop) of unoxidized actin forms (left panel). Mical-induced oxidation of M44/M47 (indicated by the double-bonded oxygen in the D-loop) suppresses the subtilisin cleavage at that site (right panel, Mox-actin). (**B**) All actin forms used in this study can be effectively oxidized by dMical (see Section 4). Cleavage of unoxidized (Unox) and Mox-actin (Mox) (2.5 μM) was detected on a 12% SDS PAGE gel at the 25 min (25) time point from subtilisin addition (0). Muscle actin forms (top panel): rabbit skeletal α-actin (RSA), bovine cardiac actin (Cardiac), and smooth muscle actin (SMA). Cytoplasmic actin forms: human β/γ cytoplasmic actin, endogenous mix, 85% β-actin (β/γ), and *Saccharomyces cerevisiae* cytoplasmic actin (Yeast).

We set out to compare the properties of cytoplasmic and muscle actins upon Mical-induced oxidation. First, we determined whether our oxidation protocol (see Section 4) is applicable to different actins. For each actin form, Mical-induced oxidation was tested by limited proteolytic digestion with subtilisin [16]. This assay is based on the observation that under limited proteolysis conditions (very low concentration of enzyme), subtilisin cleaves unoxidized (but not Mical-oxidized) actin between residues 47 and 48 in the DNAase I-binding loop, hence allowing the quantification of Mical-induced oxidation of actin (Figure 2A). As shown in Figure 2B, upon Mical-induced oxidation (“Mox”) and in no-subtilisin control samples (“0”), actin remains intact (Mw~42 kDa); however, unoxidized actin gains higher mobility in SDS PAGE gels due to the cleavage of the N-terminal part of its sequence. These results (Figure 2) validate that our oxidation protocol was effective for all actin forms in question yielding Mox-actin preparations.

**Figure 3:**
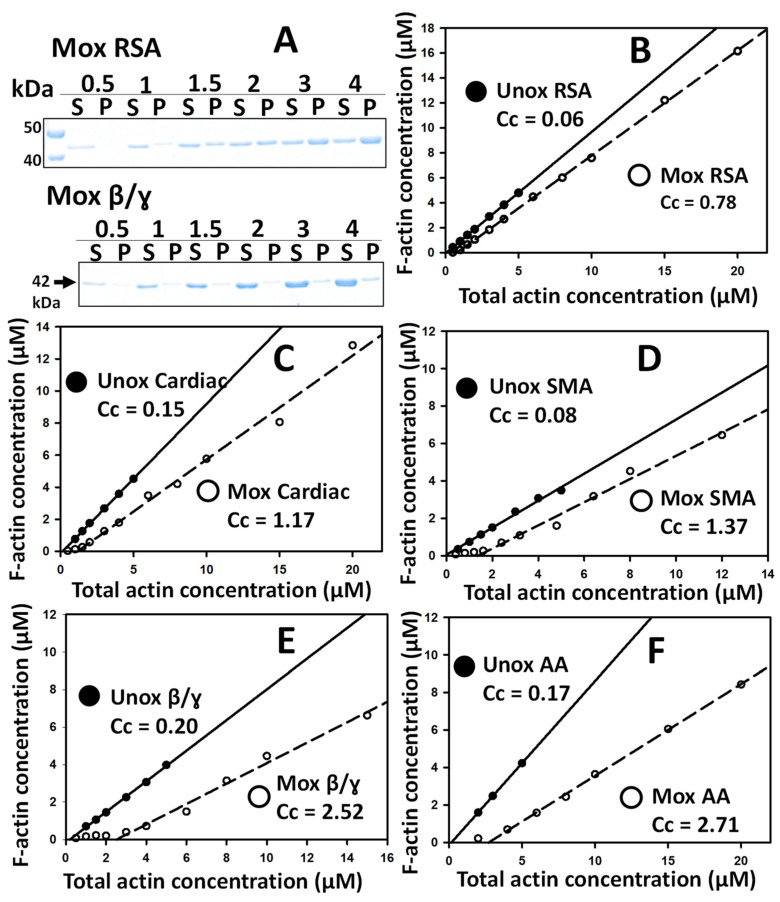
The critical concentration values of Mical-oxidized actin isoforms are indicative of the differences in their dynamic properties. (**A**) Mical-induced oxidation changes the monomer/polymer (G-/F-) distribution of Mox-RSA (top) and Mox β/γ (bottom) actins at steady state. Note the greater amounts of actin in supernatants (G-actin) in Mox β/γ samples compared to Mox-RSA. High-speed supernatants (S) and pellets (P) were analyzed by SDS PAGE and stained with Coomassie Blue. [Mox-actin] = 0.5–4 µM (displayed above the gel panels). (**B**–**F**) Determination of the Cc values of unoxidized (closed symbols) and Mox-oxidized (open symbols) actin forms. F-actin concentration vs. total actin concentration was plotted and fit as a linear curve (unoxidized actins (Unox, solid lines) and Mox-actins (Mox, dashed line)). The intersections of the linear plots with the abscissa yield the value of the Cc. The Cc values (µM) in the figure panels are the ones obtained for the displayed data sets. The average Cc values and the number of repeats are summarized in Table 1. The panels are labeled as follows: (**B**) RSA: rabbit skeletal α-actin; (**C**) Cardiac: bovine cardiac actin; (**D**) SMA: smooth muscle actin; (**E**) β/γ: human β/γ cytoplasmic actin; (**F**) AA: *Acanthamoeba castellanii* cytoplasmic actin.

To quantitatively describe the changes in the properties of different Mox-actin forms, we assessed their critical concentration (Cc) values. The Cc is a ratio of the rate constants of the actin monomers’ dissociation from and association with the ends of the filament, and it can serve as an indicator of changes in actin’s intrinsic dynamics and its propensity to polymerize. Specifically, actin will not form filaments (polymers, F-actin) below its Cc under polymerizing conditions. Mical-induced oxidation was previously shown to affect the polymerization properties of skeletal muscle actin, resulting in an increase in its Cc by an order of magnitude [15,16]. Thus, this parameter can be used to quantitatively describe potential differences in the intrinsic properties of different actin forms upon their oxidation by MICALs. To this end, we employed fully Mical-oxidized actin preparations from which residual NADPH and the Mical enzyme were removed. To determine the Cc values, we employed a high-speed sedimentation assay, which was successfully used for skeletal muscle Mox-actin in prior work [15,16]. Under the conditions of high-speed centrifugation, actin monomers remain in the supernatant, while actin filaments are compacted in the pellet, allowing the quantification of the G-/F-actin distribution by SDS PAGE (Figure 3A).

First, we determined the Cc values of unoxidized actin forms and compared them to the published Cc values (Figure 3B–F, closed symbols). Unoxidized cytoplasmic and skeletal muscle α-actin had Cc values close to those previously reported [16,26,27,28,29,30,31]. Our cardiac actin preparations appeared to have an improved polymerization propensity (compared to previously reported Cc data), which likely stems from differences in the experimental conditions [32,33].

Next, we sought to determine the effects of Mical-induced oxidation on the intrinsic properties of muscle actin isoforms (as reflected by their Cc values). Upon Mical-induced oxidation, all muscle actin forms (α-skeletal (RSA), cardiac, and γ-smooth (SMA)) exhibited an increase in their Cc compared to their unoxidized counterparts (Figure 3B–D, Table 1). In our assays, the Cc of Mox-RSA, the only previously characterized Mox-actin form, matched closely to the one reported (~1.1 µM [16]). In our experiments, all muscle actins were demonstrated to increase in Cc by an order of magnitude (50–15-fold) upon Mical-induced oxidation (Figure 3B–D, Table 1). Upon Mical-induced oxidation, RSA and cardiac actin demonstrated similar Cc values, 1.00 ± 0.275 μM and 1.16 ± 0.076, respectively. The Cc of Mox-SMA was slightly higher, 1.37 ± 0.145 μM (N = 3), compared to Mox-RSA and Mox-cardiac actins. Note that SMA preparations contain ~20% of the β-cytoplasmic actin, which raises the question of whether its properties upon Mical-induced oxidation contribute to the increased Cc of Mox-SMA compared to Mox-RSA.

Thus, we next examined the properties of cytoplasmic actin forms upon Mical-induced oxidation. For these experiments, we utilized an endogenous mix of cytoplasmic β/γ-actin isoforms (85/15%). Like its muscle counterparts, β/γ-actin demonstrated an increase in Cc upon oxidation (Figure 3E). Interestingly, Mical-oxidized β/γ-actin had a ~2.5-fold higher Cc than Mox-RSA (Table 1), suggesting a greater impact of M44/M47 oxidation on cytoplasmic actins’ dynamics compared to the muscle actin isoforms. To further test this idea, we asked whether there is a similar trend with the divergent cytoplasmic actins from *Acanthamoeba castellanii* (AA)—one of the most used model cytoplasmic actin forms. In excellent agreement with our data on Mical-oxidized β/γ-actin, we documented a ~2.7-fold increase in the Cc of Mox-AA compared to Mox-RSA (Figure 3F, Table 1). In sum, our data show that Mical-mediated oxidation influences the intrinsic properties of all of the actins used in this work (Figure 3, Table 1). Specifically, this oxidation decreases their ability to polymerize, which is evident from the increase in the Cc of all of the Mox-actins studied. Our results indicate that cytoplasmic actins are more severely impacted by Mical-induced oxidation compared to muscle actins: the Cc values of their Mox forms are 2.5–2.7-fold higher than those of Mox-RSA or Mox-cardiac actins.

### 2.2. The Isoform Composition Affects Actin’s Potential as an Activator of NADPH Oxidase Activity of MICALs

F-actin serves as a substrate of MICALs, as well as an activator of the NADPH oxidase activity of these enzymes. The observed difference in Cc values for cytoplasmic and muscle actin forms indicate that their dynamics (depolymerization and/or assembly rate constants) are non-equally affected by Mical-induced oxidation. Thus, we set out to investigate whether isoform-dependent differences in intrinsic dynamics of F-actin upon Mical-induced oxidation affect its potential as an activator of Mical’s NADPH oxidase activity. To this end, we employed a validated NADPH consumption assay that mirrors the H_2_O_2_ generation patterns in our system [19,20]. We hypothesized that the differences in the intrinsic dynamics of actin isoforms may translate into different patterns of NADPH consumption by Mical and, therefore, H_2_O_2_ generation. To mimic biological conditions more closely, we utilized unoxidized actin forms and human Mical-1 (hMical-1) because it is widely expressed in many types of human cells. We focused on the comparison of actin isoforms that exhibited the most differences in intrinsic properties upon Mical-induced oxidation—RSA and cytoplasmic β-actin. To compare homogeneous preparations, we recombinantly expressed and purified human cytoplasmic β-actin from *Pichia pastoris* yeast [34]. We polymerized unoxidized RSA and human cytoplasmic β-actin isoforms and tested them as activators of NADPH consumption by hMical-1.

**Figure 4:**
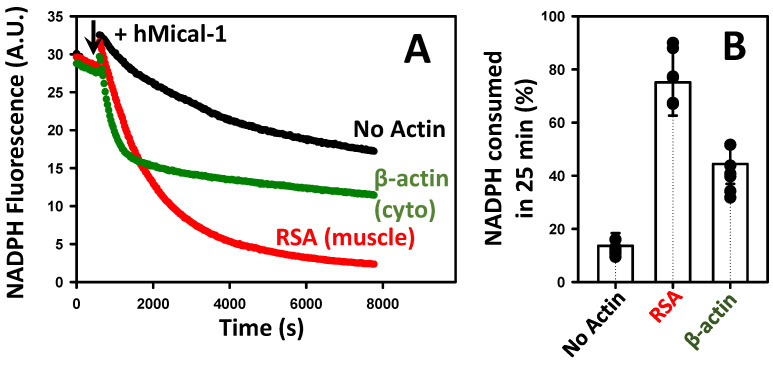
Cytoplasmic and muscle actins activate Mical-mediated NADPH consumption with different efficiencies. The time-dependent decrease in the fluorescence signal of NADPH is due to its conversion into its oxidized (non-fluorescent) form (NADP+) by Mical enzymes, which is accelerated by F-actin. The traces are color-coded as follows: hMical/NADPH only (no actin) (black triangles), RSA (red circles), and recombinant β-actin (green triangles). Note that different NADPH consumption patterns were observed in the presence of cytoplasmic vs. muscle F-actin. The addition of hMical-1 is indicated by an arrow. Excitation and emission wavelengths were set at 340 nm and 460 nm, respectively. Conditions: [Actin] = 1.5 μM, NADPH = 137 μM, and hMical-1 = 50 nM; N = 4 separate experiments. A representative data set is shown. Each trace corresponds to the average of two technical replicates (two samples) analyzed in the representative experiment shown in the figure. Two different preparations of the recombinant β-actin were tested with similar results. (**B**) Analysis of the NADPH consumption assays shown in (**A**). Note that a greater extent of NADPH consumption was observed with muscle (compared to the cytoplasmic) actin at the 25 min time point. Error bars correspond to the standard deviation.

Our results show that RSA and cytoplasmic β-actin yielded different patterns of NADPH consumption by hMical-1 (Figure 4A, compare green and red traces). Quantification of these data showed that the extent of NADPH consumption was greater in RSA-containing samples compared to cytoplasmic β-actin: ~70% vs. 40% of total NADPH, respectively, was consumed after 25 min (Figure 4B). We also observed slightly higher initial rates of NADPH consumption by hMical-1 in the presence of cytoplasmic β-actin compared to RSA (Figure 4A). Although we do not have an explanation for this observation, it may potentially reflect the differences in the rates of Mical binding to or oxidation of different actin forms. Importantly, our results suggest that the actin isoform composition itself could influence local H_2_O_2_ levels upon Mical activation.

### 2.3. Mox-Actin Can Activate NADPH Oxidase Activity of MICALs

**Figure 5:**
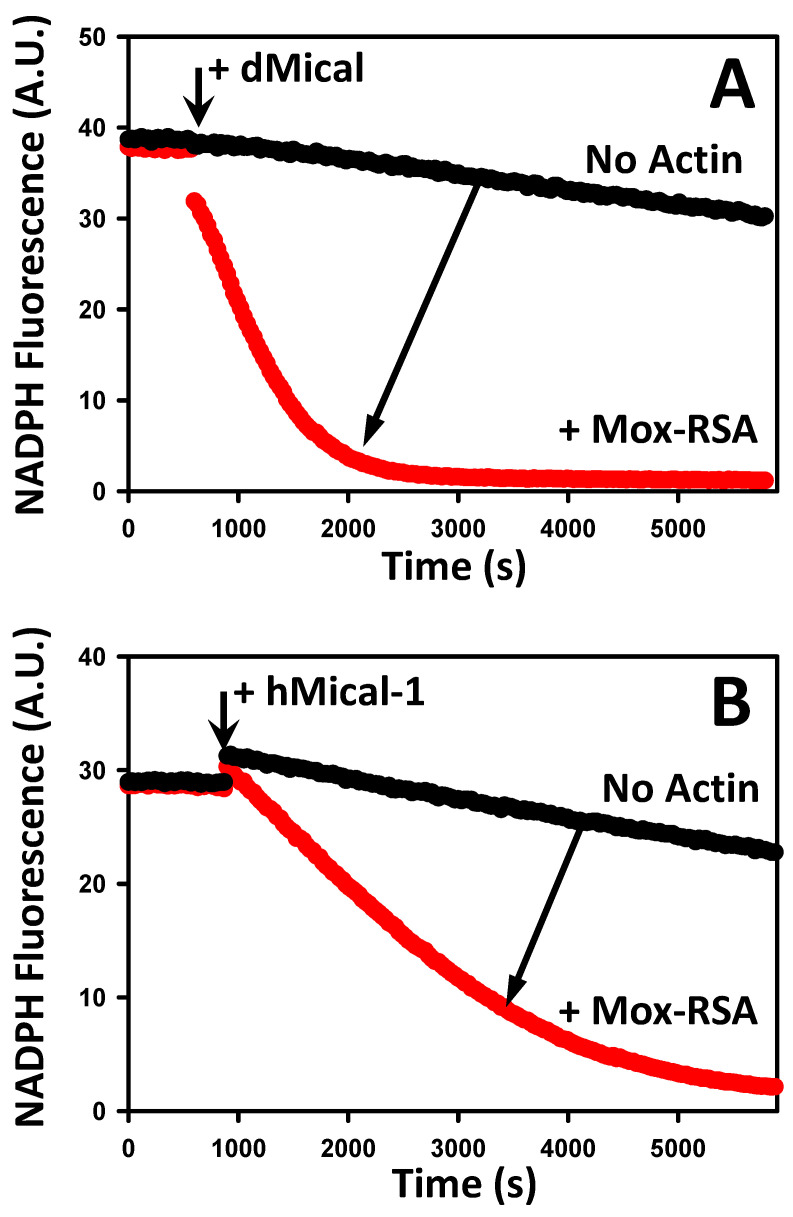
Mox-actin actin filaments are able to activate the NADPH oxidase activity of MICALs. (**A**,**B**) Mox-RSA activates the NADPH oxidase activity of dMical and hMical-1 (red traces) compared to no-actin controls (black traces, direction of the effect is shown with arrows). Representative data sets are shown, N = 3 separate experiments. Two technical replicates per condition were analyzed in each experiment. Conditions: [Mox-RSA] = 3 μM, [NADPH] = 137 μM, [dMical] = 200 nM, and [hMical] = 50 nM. Excitation and emission wavelengths were set at 340 nm and 460 nm, respectively.

High cellular concentrations of actin and the ability of Mox-actin isoforms to polymerize (Table 1) suggest that Mox-F-actin is biologically relevant. However, a connection between the redox state of filamentous actin and its potential to activate the NADPH oxidase activity of MICALs is not well established. To clarify this, we employed RSA as the best-studied isoform. We uncoupled the NADPH oxidase and monooxygenase activities of Mical by utilizing fully oxidized actin filaments (M44-SO/M47-SO) (Figure 5A). Next, we re-polymerized Mox-RSA and monitored the NADPH consumption upon the addition of NADPH and a fresh portion of the Mical enzyme. As expected, in the absence of F-actin, Mical showed a basal level of activity (Figure 5A, black trace). Importantly, we documented that in the presence of filamentous Mox-RSA, the NADPH oxidase activity of dMical increased ~15-fold (Figure 5A). To rule out the possibility that this effect stems from the heterologous actin–Mical pairing (mammalian actin/dMical), we also performed these experiments with hMical-1. Likewise, we found that the addition of Mox-RSA stimulates the NADPH oxidase activity of hMical-1 (Figure 5B, compare red and black traces). Thus, our results unambiguously show that actin, which was fully oxidized by Mical enzymes, can still serve as an activator of their NADPH oxidase activity.

### 2.4. Actin-Binding Factors Fine-Tune the Capacity of Mox-Actin to Activate NADPH Oxidase Activity of MICALs

In cells, actin exists in complexes with a variety of regulatory factors. Mox-actin retains its ability to interact with some of the key actin regulators [16,18,35] and can activate the NADPH oxidase activity of MICALs (Figure 5). Thus, we asked whether NADPH oxidase activity can be fine-tuned by filaments’ stabilizing and destabilizing factors. To uncouple the NADPH oxidase and monooxygenase activities of Mical, we utilized fully oxidized actin filaments. Next, we manipulated the stability of Mical-oxidized RSA by employing previously validated tools, namely, the mushroom toxin phalloidin (universal F-actin stabilizer), human cofilin-1, and swinholide A (actin destabilizing factors) [36,37,38,39,40].

**Figure 6:**
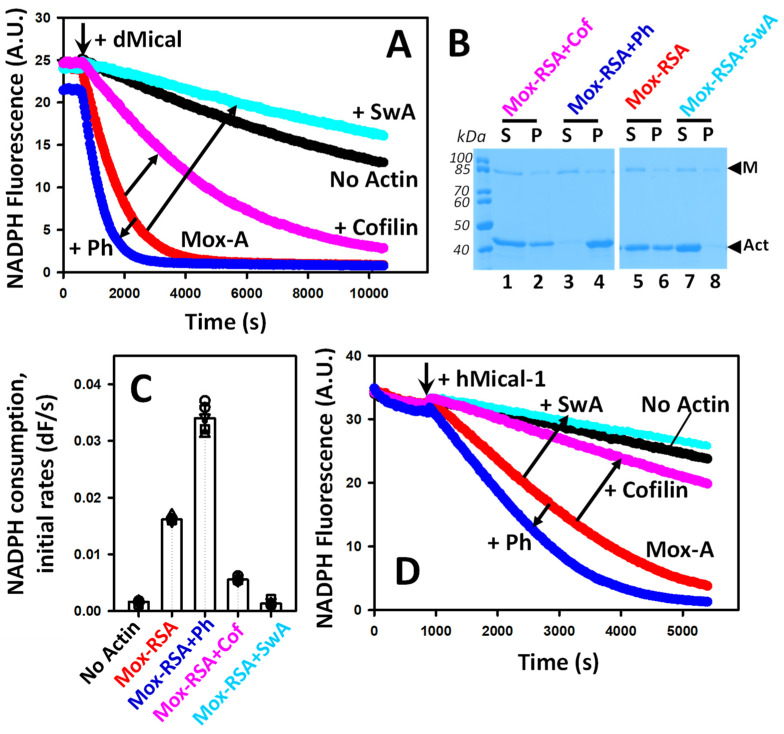
Interactions of Mox-actin with stabilizing and destabilizing factors fine-tune the NADPH oxidase activity of MICALs. (**A**) The effects of F-actin-stabilizing and destabilizing agents on NADPH consumption by dMical in the presence of Mox-actin filaments. An F-actin stabilizer (phalloidin) enhances the NADPH oxidase activity of dMical, and F-actin destabilizers (hCofilin-1 and swinholide A) inhibit it. The addition of dMical is indicated by an arrow. The traces are labeled as follows: Mical/NADPH only, no actin (black, n = 8 samples); Mox-RSA (red, n = 8 samples); Mox-RSA + phalloidin (Ph, dark blue, n = 8 samples); Mox-RSA + hCofilin-1 (Cofilin, magenta, n = 8 samples); and Mox-RSA + swinholide A (SwA, light blue, n = 4 samples). A representative data set is shown. Each trace corresponds to the average of two technical replicates (two samples) analyzed in the representative experiment shown in the figure. (**B**) Determination of the G-/F-Mox-actin content in the reactions monitored in (**A**). The samples were subjected to high-speed centrifugation at the end of the observation time. The supernatants (S) and pellets (P) for each condition were analyzed by SDS-PAGE, stained with Coomassie Blue, and analyzed using Fiji 2.0 software. The bands corresponding to actin and Mical are denoted by “M” and “Act”, respectively. (**C**) Analysis of the initial rates of the NADPH consumption by dMical in the presence of F-actin-stabilizing and -destabilizing molecules, as shown in panel A. The initial rates were determined from the linear portions of the NADPH consumption curves (first 600–1000 s). Error bars correspond to the standard deviation. (**D**) The effects of F-actin-stabilizing and destabilizing molecules on NADPH consumption by hMical-1 in the presence of Mox-RSA. The addition of hMical-1 is indicated by an arrow. The traces are labeled and color-coded the same as in A for clarity of presentation. A representative data set is shown. Each trace corresponds to the average of two technical replicates (two samples) analyzed in the representative experiment shown in the figure (N = 3 separate experiments, 6 samples total). Conditions: [Mox-RSA] = 3 μM, [NADPH] = 137 μM, [dMical] = 200 nM, [hMical-1] = 50 nM, [phalloidin] = 3.3 μM, [hCofilin-1] = 300 nM, [swinholide A] = 1.5 μM. Excitation and emission wavelengths were set at 340 nm and 460 nm, respectively.

We documented that upon the addition of phalloidin-stabilized Mox-RSA, dMical consumes NADPH faster than in the presence of uncomplexed Mox-RSA (Figure 6A, dark blue trace). On the other hand, hCofilin-1 reduces the initial rates and extent of NADPH consumption by dMical (Figure 6A, magenta trace), as previously reported with unoxidized F-actin preparations [16]. Pelleting assays confirmed that when Mox-RSA was stabilized with phalloidin, more F-actin was present in the samples compared to the unstabilized Mox-RSA (Figure 6B, pelleted F-actin denoted as “P”). On the other hand, hCofilin-1 addition resulted in only a minor decrease in F-actin in the system compared to Mox-RSA (Figure 6B, compare lines 2 and 6). Overall, our data show that the stabilization of Mox-F-actin enhances NADPH consumption by Mical and vice versa. Since cofilin was previously found to be a redox-sensitive protein, whose activity could be potentially altered by the H_2_O_2_ generated in our system, we sought additional confirmation of the results suggesting that F-actin destabilization inhibits NADPH consumption by Mical. To this end, we employed the previously characterized macrolide toxin swinholide A (SwA) to destabilize Mox-F-actin (Figure 6A,C) [41]. The addition of swinholide A reduced dMical’s NADPH oxidase activity to the basal level—similar to that observed in the absence of Mox-F-actin (Figure 6A, light-blue trace). The effect of SwA on the NADPH oxidase activity of Mical was greater than that of hCofilin-1 (Figure 6C). This is in line with the results of the pelleting assays, which revealed that practically all Mox-F-actin was converted to its monomeric form in the presence of SwA, but with hCofilin-1, Mox-F-actin can still be detected (Figure 6B). Thus, the destabilization of Mox-actin by SwA showed the same trend as observed with cofilin—the inhibition of NADPH consumption by Mical. The analysis of the initial rates and extents of NADPH consumption under different conditions is shown in Figure 6C. To determine whether the same trend stands for the mammalian actin/Mical pair, we performed NADPH consumption assays with hMical-1 (Figure 6D). Similar to our results with dMical, phalloidin-stabilized Mox-RSA showed greater activation of the NADPH oxidase activity of hMical-1 compared to Mox-RSA alone. Likewise, the addition of hCofilin-1 or SwA diminished Mox-actin’s potential as an activator. Thus, actin-binding factors can alter the potency of Mox-F-actin as an activator of Mical’s NADPH oxidase activity.

## 3. Discussion

### 3.1. Toward Uncovering the Role of Actin Isoforms

Our understanding of the differential regulation of actin isoforms is highly incomplete and represents a large gap in knowledge. However, more examples of it are starting to emerge. Among the actin-binding proteins that have demonstrated actin-isoform-related preferences are the formin family proteins (delphilin and DIAPH3 (mDia2)) [5,42], profilin, and thymosin-β4 [2]. Likewise, N-terminal arginylation occurring in cytoplasmic β-actin (but not γ-actin) negatively affects actin nucleation by Arp2/3 and formin-driven filaments’ elongation [43]. Importantly, this study establishes redox modification by Mical enzymes as a new example of isoform-dependent actin regulation.

### 3.2. Actin Isoform Composition as a Novel Factor Influencing the Regulatory Impact of Mical Enzymes

The functional output of Mical activation can be termed the redox regulation of primary and secondary targets to exert a specific cellular function. Such output would be defined by a combination of several factors. Firstly, MICALs are activated as per cellular needs downstream of the Semaphorin–Plexin pathway, and their activation time window can be fine-tuned by Rab GTPases and other factors [6]. Thus, the spatiotemporal patterns of Mical activation would influence cellular outcomes. Secondly, NADPH levels would depend on the cell type and specific conditions [44]. Thirdly, the expression and activation levels of the proteins that synergize with Mical-mediated redox regulation (MsrB, cofilin, profilin, and formins [16,18,45,46]) may also vary depending on the cell type and stage of development. Importantly, this work identifies the actin isoform composition as a novel factor that affects the functional outcomes of Mical activation. Specifically, we show that F-actin oxidation by Mical affects its intrinsic properties in an isoform-dependent manner. We demonstrate that the Cc values for cytoplasmic Mox-actins are higher than those of muscle Mox-actins (Table 1), suggesting that upon the activation of MICALs, actin networks built from cytoplasmic actin isoforms may be destabilized more readily than those built from the muscle isoforms. One of the open questions to be addressed in future studies is how F-actin’s isoform composition and decoration by actin-binding proteins would affect the rate and extent of oxidation of actin-based structures. In sum, our results suggest that the isoform composition itself may affect the dynamics of actin networks upon Mical activation.

MICALs are unique enzymes that function as monooxygenases using actin filaments as their main substrate, as well as NADPH oxidases, which are activated in the presence of F-actin [12,20]. In this study, we uncovered a previously unknown connection between the actin isoform composition and the NADPH oxidase activity of MICALs. Strikingly, we found that F-actins built from different isoforms (alpha-skeletal muscle vs. beta-cytoplasmic) have different potential as activators of MICALs’ NADPH oxidase activity. Specifically, we observed a greater extent of NADPH consumption with RSA compared to the cytoplasmic beta-actin. This could be attributed (at least in part) to differences in their dynamic parameters as actin isoforms undergo Mical-induced oxidation (Table 1). Our data are consistent with the idea that activated MICALs can produce distinct H_2_O_2_ generation patterns (mirroring NADPH consumption) depending on the isoform composition of local actin networks. We speculate that this may result in different levels of redox regulation of secondary protein targets by locally emitted H_2_O_2_.

### 3.3. Mox-Actin as an Activator of the NADPH Oxidase Activity of MICALs

Prior to this work, it was not definitively proven that Mox-actin can serve as an activator of its NADPH oxidase activity. However, the published observations are consistent with such a scenario since Mical enzymes are able to oxidize amounts of NADPH that largely exceed the amounts of F-actin present in the reaction [10,19,20]. Our data unambiguously show that filaments formed exclusively from M44-SO/M47-SO subunits activate NADPH consumption by MICALs. Such activation was observed with human and fly Mical orthologs, suggesting that these results represent a general trend. These findings are biologically relevant since all of the actin forms examined in this work can exist in a Mox-actin state and, therefore, could persist in cells. Thus, on a cellular level, actin networks fully oxidized by Mical would have the potential to activate NADPH consumption, resulting in H_2_O_2_ generation.

### 3.4. The Stabilization and Destabilization of Mox-Actin Modulates the NADPH Oxidase Activity of MICALs

Our results link the effects of the stabilization and destabilization of Mox-F-actin to the fine-tuning of Mical’s NADPH oxidase function. In this work, we utilized the most studied MICALs from different organisms (fly and human) and observed the same trends, which makes our conclusions widely applicable. We found that phalloidin-stabilized Mox-RSA activated NADPH consumption by Mical enzymes better than unstabilized Mox-RSA filaments. This can be at least in part attributed to the preservation of Mox-actin filaments since only filamentous and not monomeric actin serves as an activator of MICALs’ NADPH oxidase activity (Figure 6B). Additionally, phalloidin binding to F-actin may influence its interaction with Mical, which is yet to be investigated. On a cellular level, Mox-actin networks could be stabilized by actin-bundling and side-binding proteins. Although one of the recent studies documented that tropomyosin 1.8 does not protect F-actin from Mical-induced disassembly [17], it should be noted that close to 40 tropomyosin isoforms are known, and their effects on Mox-F-actin are yet to be tested [47].

Likewise, actin-destabilizing factors (cofilin and swinholide A) lowered the rate and extent of NADPH consumption. Interestingly, in the presence of Mox-actin and swinholide A (SwA), NADPH consumption was slower than in the no-actin control, suggesting that SwA may be an uncharacterized inhibitor of Mical. As previously observed [16], we found that the presence of cofilin strongly suppresses Mical’s NADPH oxidase activity compared to Mox-RSA, but it was still enhanced compared to the no-actin control. At the same time, the pelleting assay indicated that similar amounts of F-actin were present in Mox-RSA samples with and without cofilin. We speculate that in addition to the extensive severing [16], cofilin may hinder Mical’s interaction with Mox-filaments and slow down NADPH consumption. Although previous work documented that Mical and cofilin binding to unoxidized actin is not mutually exclusive [16], the mode of cofilin binding to unoxidized and Mox-F-actin is yet to be determined. Alternatively, extensive cofilin-mediated severing of Mox-F-actin may generate very short actin filaments, which may have a reduced ability to activate MICALs compared to longer filaments. Our data suggest that in cells, the factors modulating F-actin stability (or those competing with MICALs for F-actin binding) could fine-tune the activities of these enzymes.

In sum, our data suggest that the actin isoform composition and binding factors fine-tune the functional output of Mical enzymes.

## 4. Materials and Methods

### 4.1. Protein Expression, Purification, and Handling

Rabbit Skeletal Actin (RSA, identical to human) was purified from acetone powder (Pel FREEZ, 41995-2) [48]. *Saccharomyces cerevisiae* cytoplasmic (yeast) actin was prepared employing DNAase I affinity chromatography followed by anion exchange [49]. Lyophilized actin isoforms and physiological mixes were purchased from Cytoskeleton Inc.: bovine cardiac muscle actin (AD99-B identical to human); human endogenous cytoplasmic β/γ-actin mix (APHL99-E, β/γ-actin, 85/15%); and chicken gizzard smooth muscle (SMA) actin mix (AS99-A, γ-smooth/β-cytoplasmic, 80/20%). All of the lyophilized actins used in this study were subjected to one round (cycle) of polymerization/depolymerization prior to the experiments. Specifically, actins were reconstituted in S200 buffer (2 mM Tris pH 8, 0.2 mM CaCl_2_, 0.25 mM TCEP, and 0.01% NaN_3_, supplemented with 0.2 mM ATP and 1 mM DTT) and incubated on ice for 1 h (hr), sonicated for 5 min in an ice-cold water bath, and then incubated overnight on ice. Actin preparations were centrifuged for 15 min at 14,000 rpm, 4 °C (FA-45-30-11, Eppendorf, Hamburg, Germany), and the resulting supernatants were polymerized for 1 h at room temperature (RT) in S200 buffer supplemented either with 100 mM KCl (muscle actin forms) or with 100 mM KCl and 1 mM MgCl_2_ (cytoplasmic forms). F-actin was pelleted at 54,000 rpm, 4 °C for 30 min, in an MLA80 rotor (Beckman Coulter, Brea, CA, USA). The pellets were soaked in S200 buffer for 2 h on ice and then dialyzed against buffer GB2 (2 mM Tris, 0.2 mM CaCl_2_, 0.2 mM ATP, 2 mM β-mercaptoethanol) supplemented with 0.01% NaN_3_ with one buffer change. The resulting actin preparations were spun down at 54,000 rpm, 4 °C for 30 min, in an MLA-130 rotor (Beckman Coulter), and monomeric actin was recovered in supernatants. All actin preparations were stored on ice.

Human recombinant β-actin was expressed in *Pichia pastoris* yeast as a chimeric protein (N-terminal ubiquitin fusion and C-terminal thymosin-β4 peptide followed by a poly-histidine tag) (strain MBY12639 was kindly provided by Dr. Mohan K. Balasubramanian, University of Warwick, UK) [34]. Cells were inoculated into YPD media (2% tryptone, 1% yeast extract, 1% glycerol, and 100 µg/mL of zeocin (Invitrogen, 46-0509, Waltham, MA, USA)), and were cultured at 30 °C overnight. The starting culture was diluted in BMG media (0.34% Yeast Nitrogenous Base (BD Difco, 233520, Franklin Lakes, NJ, USA), 1% ammonium sulfate, 100 mM potassium phosphate buffer (pH 6), 1% glycerol, 0.00004% biotin), and cells were cultured at 30 °C until OD600 reached ~1.5. Next, the media were switched to BMM (0.34% Yeast Nitrogenous Base, 1% ammonium sulfate, 100 mM potassium phosphate buffer (pH 6), 0.5% methanol, 0.00004% biotin). The cells were grown at 27 °C for 48 h with the addition of 0.5% methanol every 24 h, collected by centrifugation, resuspended in phosphate buffer (Gibco, 10010-023, Waltham, MA, USA), frozen in liquid nitrogen, and stored at −80 °C. To purify the protein, cells were powderized in liquid nitrogen and mixed with one volume of 2× lysis buffer (20 mM Imidazole, 20 mM Hepes pH 7.4, 600 mM NaCl, 4 mM MgCl_2_, 2 mM ATP, 1 mM PMSF, 1µg/mL leupeptin hemisulfate, 1 µg/mL pepstatin A). The cells were lysed in a BeadBeater using 0.5 mm glass beads (10 times, 30 s with 3.5 min intervals) and then sonicated 4 times at 50–60% power. The lysates were precleared by centrifugation at 16,000× *g* for 45 min at 4 °C (50.2Ti rotor, Beckman Coulter) and then filtered through a 0.45 µM syringe filter. The lysates were loaded onto a 2 mL HisPur Ni-NTA gravity column (Thermo Scientific, 88221, Waltham, MA, USA). The resin was washed with a low-imidazole buffer (10 mM imidazole, 20 mM HEPES pH 7.4, 300 mM NaCl, 2 mM MgCl_2_, 1 mM ATP), followed by a low-salt buffer wash (5 mM HEPES pH 7.4, 0.2 mM CaCl_2_, 0.2 mM ATP, and 0.5 mM dithiothreitol (DTT)). Actin was cleaved on the column with 5 µg/mL chymotrypsin (Sigma Aldrich, C3142, St. Louis, MO, USA) for 24 h at 4 °C. The cleavage reaction was stopped with 1 mM PMSF. Actin was concentrated, polymerized with 100 mM KCl, 1 mM MgCl_2_, and 10 mM DTT overnight at 4 °C, and pelleted at 54,000 rpm for 30 min at 4 °C (MLA-130 rotor, Beckman Coulter). The resulting actin pellet was soaked in S200 buffer, depolymerized on dialysis against the same buffer, and subjected to high-speed centrifugation at 54,000 rpm for 30 min at 4 °C (MLA-130 rotor). *Acanthamoeba castellanii* cytoplasmic actin (AA) was a kind gift from Prof. Margot Quinlan (UCLA). Actin concentration was determined by quantitative SDS PAGE gels employing RSA of known concentration as standard.

The DNA construct of *Drosophila melanogaster* Mical (dMical) encoding RedoxCH domains with a N-terminal Nus-tag and the N-terminal and C-terminal 6xhistidine tags in ampicillin-resistant pET43.1NG vector was a kind gift from Prof. Jonathan Terman (UT Southwestern Medical Center). The dMical enzyme was expressed and purified as previously described [15,16] using a combination of metal affinity and ion exchange chromatography. The lyophilized human Mical-1 (hMical-1, Cytoskeleton Inc., MIC01, Denver, CO, USA) was reconstituted in H_2_O (50 μL), incubated on ice for 1 h, mixed, aliquoted, and stored at −80 °C.

Human cofilin-1 (hCofilin-1) in the ampicillin-resistant pBAT4 vector was expressed in BL21(DE3) *E. coli* cells cultured in MMI liquid media (1.25% tryptone, 2.5% yeast extract, 125 mM NaCl, 0.4% glycerol, 50 mM Tris, pH 8.2). The cells were grown at 37 °C until reaching an OD_600_ of 1.0, followed by induction with 1 mM IPTG at 25 °C overnight. The cell pellets were stored at −80 °C. For purification, the cells were resuspended in the extraction buffer (20 mM MOPS, pH 7, 1 mM NaN_3_, 0.5 mM ethylene glycol-bis(β-aminoethyl ether)-N,N,N′,N′-tetraacetic acid (EGTA), 50 mM NaCl, 2 mM DTT, 0.5 mM PMSF) and then sonicated 7 times for 30 s at 40% power with 3 min cooldown intervals. The lysate was clarified by centrifugation and syringe-filtered (0.22 μm). The lysate was nutated with 20 mL of anion exchange resin (IonSep DEAE Cellulose (DE52); Biophoretics Inc., Sparks, NV, USA) for 1 h at 4 °C. hCofilin-1 was recovered in the flow-through and applied to a 5 mL HiTrap SP FF column (Cytiva, Marlborough, MA, USA). The column was washed with a no-salt buffer (20 mM MOPS, pH 7, 1 mM NaN_3_, 0.5 mM EGTA, 2 mM DTT, 0.5 mM PMSF), and the protein was eluted with a 0–100% gradient of a high-salt buffer (20 mM MOPS, pH 7, 1 mM NaN_3_, 0.5 mM EGTA, 700 mM NaCl, 2 mM DTT, 0.5 mM PMSF) in 10 column volumes. Cofilin was concentrated and dialyzed against the storage buffer (10 mM MOPS, pH 7, 25 mM NaCl, 0.1 mM PMSF) overnight at 4 °C. The protein concentration was determined using the extinction coefficient 14,440 at 280 nm (with a baseline at 350 nm).

### 4.2. Preparation of Mical-Oxidized (Mox-) Actin Forms

Actin was dialyzed against G-Buffer-5 (GB5; 5 mM Tris pH 8, 0.2 mM CaCl_2_, 0.2 mM ATP, and 0.5 mM DTT) overnight at 4 °C. The next day, actin was polymerized with 2 mM MgCl_2_ and 50 mM KCl for 1 h at RT. F-actin (10 μM) was oxidized for 10 min at RT with the addition of dMical (100 nM) and NADPH (300 μM). Mox-actin was dialyzed against GB2 overnight and centrifuged (MLA130, 20 min, 77,000 rpm, 4 °C) to separate the supernatant containing monomeric Mox-actin. To remove the Mical enzyme, Mox-G-actin was mixed with 250 μL of settled Ni2+ resin equilibrated with GB2 and nutated at 4 °C for 40 min. The flow-through containing Mox-actin was collected and dialyzed against S200 buffer overnight at 4 °C. Mox-actin was spun down (MLA-130 rotor, 20 min, 77,000 rpm, 4 °C) to pellet any aggregates. The concentration was determined in a Bradford assay using RSA of known concentration as a standard.

### 4.3. Determination of Critical Concentration (Cc) of Actin Forms

Mg-ATP-G-actin (unoxidized or Mox-) was obtained by incubation with 100× exchange buffer (0.05 mM MgCl_2_ and 0.2 mM EGTA, final concentrations) for 3 min at RT. Actin was polymerized for 1 h at RT in KMEH buffer (10 mM HEPES pH 7, 10 mM EGTA, 10 mM MgCl_2_, 500 mM KCl) supplemented with 1 mM DTT and 0.2 mM ATP. Actin was diluted in the same buffer, followed by overnight incubation at 4 °C. To quantify the monomer/polymer distribution, samples were subjected to high-speed centrifugation (TLA100, 20 min, 90k rpm, 4 °C). Supernatants and pellets were analyzed by SDS-PAGE alongside the Unstained Protein Ladder (Thermo Scientific, PI26614). Gels were stained with Coomassie blue and imaged using a GelDoc EZ System (Biorad, 1708270, Hercules, CA, USA). The band intensities of actin in supernatants (S; G-actin) and pellets (P; F-actin) were determined using Fiji 2.0 software [50]. For each sample, the total intensity was defined as the sum of band intensities of the pellet and the supernatant. The intensity values were related to the total actin concentration. The Cc values were calculated from the x-intercepts of linear [F-actin] vs. [Total actin] plots [15,16].

### 4.4. NADPH Consumption Assay

Mox-actins were polymerized in KMEH buffer supplemented with 0.2 mM ATP and 1 mM DTT for 1 h at RT. For some experiments, F-actin samples were stabilized with phalloidin (Sigma Aldrich, 50-175-1435) at a 1:1.1 (actin–phalloidin) molar ratio. Actin was diluted in S200 buffer supplemented with KMEH and incubated for 17 h at 4 °C. The next day, F-actin samples were supplemented with NADPH. Fluorescence was monitored using a Varioskan Flash plate reader (Thermo Scientific) equipped with SkanIT 2.2 software. The baseline of the fluorescent signal was recorded for 10 min at 30 s intervals. Upon the addition of Mical enzymes, the decrease in the fluorescence of NADPH (reduced form) upon its conversion into non-fluorescent NADP+ was monitored (excitation, 340 nm; emission, 460 nm). In some cases, hCofilin-1, Swinholide A (AdipoGen, 50-148-9962, San Diego, CA, USA), or phalloidin was added to F-actin simultaneously with Mical, and fluorescence was monitored for 2 h with reading intervals of 30 s. The linear portions of the resulting curves were used to calculate the initial rates of NADPH consumption. The data were fit as a linear function using SigmaPlot v11 software or Microsoft Excel. The resulting slopes yielded the initial rates (dF/s).

### 4.5. Limited Proteolysis with Subtilisin

Subtilisin (Sigma, P5380-25MG, St. Louis, MO, USA) digestion of actin was used to detect actin oxidation by Mical [16,18]. The reactions were started by the addition of subtilisin at a 1:1000 subtilisin–actin *w*/*w* ratio to G-actin (2.5 μM). The reaction time was 0–30 min at RT. The subtilisin cleavage was stopped with 0.31 mM of PMSF, and the samples were analyzed by SDS-PAGE.

## 5. Conclusions

Actin is one of the most abundant proteins in eukaryotic cells, with six isoforms expressed in vertebrates. Actin is a substrate of MICALs and an activator of H_2_O_2_ generation by these enzymes. This study shows for the first time that Mical-induced oxidation destabilizes actin isoforms to a different extent. Furthermore, our results connect the intrinsic dynamics of actin isoforms and their redox state with the patterns of H_2_O_2_ generation by MICALs. Thus, the activation of MICALs may have different functional outputs in different cells and tissues depending on the chemical identities of local actin networks.

## Figures and Tables

**Table 1:** Values of critical concentration determined for different forms of unoxidized and Mical-oxidized (Mox-) actin.

Actin	Mox-Actin Cc (μM)		Unoxidized Actin Cc (μM)	
Muscle				
RSA **	1.00 ± 0.28		0.020 ± 0.048	
Cardiac *	1.16 ± 0.08		0.080 ± 0.068	
SMA *	1.37 ± 0.15		0.090 ± 0.013	
Cytoplasmic				
β/γ-actin **	2.44 ± 0.30		0.24 ± 0.18	
AA ^#^	2.71	3.91	0.17	0.10

** N = 4 separate experiments, average ± standard deviation (s.d.); * N = 3 separate experiments, average ± s.d.; ^#^ N = 2 separate experiments.

## Data Availability

The data supporting the findings of this study are available from the corresponding authors on reasonable request.
